# Supplementary material for: Dynamic characteristics and fatigue life analysis of the gyration platform of roadheader
Source: PLoS One. 2024 Dec 13;19(12):e0314351. doi: 10.1371/journal.pone.0314351 (PMC11642995; doi:10.1371/journal.pone.0314351)
Supplement: Attachment — Submitted filename: Response-to-Reviewers.doc [file pone.0314351.s001.doc]

**Original Manuscript ID:** PONE-D-23-35060

**Original Article Title:** Dynamic characteristics and fatigue life analysis of gyration platform of roadheader

**To:** PLOS ONE

**Re:** Response to reviewers

Dear Editor and reviewers,

Thank you for your letter and the reviewers’ comments on our manuscript entitled "Dynamic characteristics and fatigue life analysis of gyration platform of roadheader" (ID: PONE-D-23-35060). Those comments are very helpful for revising and improving our paper, as well as the important guiding significance to other research. We have studied the comments carefully and made corrections which we hope meet with approval. The main corrections are in the manuscript and the responds to the reviewers’ comments are as follows .

Replies to the reviewers’ comments:

**Reviewer#1**

Manuscript entitled "Dynamic characteristics and fatigue life analysis of gyration platform of roadheader" analyzed the cutting parameters of a roadheader during the excavation process. Some dynamic data was captured and discussed regarding the position and orientation or the roadheader. The authors believed that this study could provide a reference for the improvement of the gyration platform structure and the selection of a suitable swing speed.

Special thanks to you for pointing out some important modifications needed in the manuscript. We have revised the manuscript according to your kind advises .

**Concern # 1:** The methods and the tools that the authors selected to carry out the research were too rough and simple. The authors are not familiar with the real working conditions of an excavator, there are many types for different working environments, to be straight forward and simple, coal cutter is coal cutter, roadheader is roadheader, one is for long wall mining face, one is for roadway excavation. The authors need to add more details of the differences, regarding the working conditions of them, rather than mix them together, sometimes declare that the roadheader is also adaptable for long wall mining face, which is clearly not precise.

**Author response:**

Thank you very much for your comments. The roadheader is mainly used in tunnel excavation and coal mine tunnel excavation work. Based on the comments of the reviewers, we have revised the content of the introduction.

The revisions are as follows:

The expeditious and secure excavation of coal mine roadway stands as a fundamental prerequisite for guaranteeing elevated coal production [1,2]. In the demanding context of coal mine roadway excavation, the cutting heads confront a milieu of complexity and harshness. This is exacerbated by the unstable physical and mechanical attributes of the cutting target (a heterogeneous coal-rock mass).

**Concern # 2:** As stated by the authors, ‘In order to ensure the reliability of the gyration platform, when cutting in different swing directions, the swing speed should be reasonably selected based on the hardness of the coal and rock’. How did the authors consider the up and down movement, rather than just left and right, the real scenario for a roadheader is all directions, rather than just horizontal movement. Though it is acceptable to just discuss the horizontal one, it is indeed necessary to also consider the horizontal movement one, at least to add some discussions regarding the limitations of this study.

**Author response:**

Thank you very much for your comments. In the conclusion section of the manuscript, we have added some discussions and explanations on the limitations of this study in the conclusion section.

The revisions are as follows:

In this study, we concentrated on scrutinizing the horizontal rotary motion of the roadheader. Nevertheless, during the practical cutting process, the roadheader exhibits both horizontal and vertical movements. The dynamic attributes of the roadheader exhibit variations in different directions of motion. Consequently, our study possesses certain limitations within its scope. To conduct a more comprehensive analysis of the roadheader's performance throughout the entire cutting process, our subsequent research will prioritize examining the dynamic characteristics of the roadheader during vertical motion.

**Concern # 3:** In section 2.1.3, the authors stated that the cutting head load was calculated by Matlab software, but the inner process is lacking, the purpose and the results from the calculation are also unknown, which both make this section rough and insufficient in details.

**Author response:**

Thank you very much for your comments. Considering the structure and installation parameters of each pick, the force on an individual pick under specific conditions can be computed using Formulas 1-3. Subsequently, employing the calculation methodology outlined in Formulas 4-9, the external load experienced by the cutting head can be determined by converting the force on an individual pick to the center of mass of the cutting head. According to the reviewer's comments, we have added a flowchart for calculating the cutting head load. Based on this flowchart, we used MATLAB to achieve the calculation of the load.

The revisions are as follows:

In order to analyze the dynamic reliability of the roadheader, when analyzing the load of the cutting head under complex conditions, the rock samples to be excavated can be tested to obtain their physical and mechanical properties. Combined with the force analysis of the cutting pick, the cutting head load can be calculated. The calculation process is shown in Figure 4.

**Figure 4.** The flow of cutting performance calculation

**Concern # 4:** In section 2.1.3, the authors mentioned firmness coefficient, what was the strength values of the rock?

**Author response:**

We updated the manuscript by adding the property parameters of rock samples according to the suggestions provided by Reviewer #1.

The revisions are as follows:

Based on the above analysis, a program was developed to calculate the cutting head load of the roadheader. The operational parameters were determined based on the geological conditions specific to this model's mining face. The properties of rock samples cut by the given roadheader were determined, and the results of the property measurements were presented in Table 1. When calculating the load, the average of two measurement results was taken for the computation. The cutting motor power is 75 kW, and the cutting head rotation speed is set at 53 r/min. Detailed parameters of the cutting head are provided in Table 2. Operating at full load, the maximum transverse swing speed of the cutting head is approximately 1.1 m/min. By executing the program, the instantaneous load of the cutting head can be computed for two working conditions: transverse swing from left to right and transverse swing from right to left. The resulting cutting head load curves are illustrated in Figure 6.

Table 1. Property parameters of rock samples

| NO. | Density (kg/m^3^) | Elastic modulus (MPa) | Poisson's ratio | Tensile strength (MPa) | Compressive strength (MPa) | Firmness coefficient |
| --- | --- | --- | --- | --- | --- | --- |
| 1 | 2610 | 18300 | 0.22 | 5.24 | 52 | 6.1 |
| 2 | 2620 | 17500 | 0.21 | 5.11 | 49 | 5.9 |

**Concern # 5:** Also in section 2.1.3, how did the authors obtain the data? it is a necessity to briefly introduce the methods, or the arrangement of the sensors that captured all data.

**Author response:**

Thank you very much for your comments, and we have added a description of the data collection in the experiment and revised Figure 9 (Revised as Figure 10).

The revisions are as follows:

The torque during the cutting process was obtained by installing a torque sensor on the cutting motor torque shaft, and the torque value was obtained using a data acquisition device.

Figure 10. Cutting test

**Concern # 6:** The most serious flaw of this paper exists in Fig. 8, obviously the cutting test couldn't shape a rectangular groove as shown in the figure, the cutting head was an overall domed appearance, how did it possible for a domed head cut a rectangular groove?

**Author response:**

Thank you very much for your comments. This experiment was conducted at the Mining Hydraulic Technology and Equipment Engineering Research Center, equipped for testing and analyzing the dynamic performance of mining machinery, including shearers and roadheaders, as shown in Figure (a) . The simulated coal wall employed in this study serves for cutting experiments with roadheaders, as well as for experiments involving the cutting of the top and bottom rock layers by coal shearers, as shown in Figure (b). The rectangular groove depicted in Figure 9 is a remnant from a prior shearer cutting test.

(a)

(b)

**Reviewer#2**

**Concern # 1:** The fatigue lifespan expression and/or result are not enough in the current manuscript that is not consist with the defined TITLE.

**Author response:**

Thank you very much for your comments. This article investigates the dynamic characteristics of the gyration platform during lateral cutting, including the stress, deformation, and fatigue life of the gyration platform under different swing directions and speeds. Different lateral directions and swinging speeds have important effects on the stress distribution and deformation of the gyration platform. Under the premise of determining the cutting object, this article establishes the correlation between the fatigue life of the gyration platform and the horizontal swing speed. If an in-depth analysis is conducted on the fatigue life of the gyration platform, it is necessary to study the effects of different rock properties, cutting head speed, horizontal swing speed, and up and down swing speed on the fatigue life of the gyration platform. Thank you very much for your comments. In future research, we will conduct a more in-depth study on the fatigue life of the gyration platform.

**Concern # 2:** The analysis reference is not referring the classical strength theory despite the manuscript filling with enough stress and strain maps.

**Author response:**

Thank you very much for your comments. We have added the classical strength theory and corresponding references.

The revisions are as follows:

According to the Miner's Rule, the load acting on the gyration platform is composed of *m* different stresses[22]. The number of cycles at each stress level is denoted as *n*_1_, *n*_2_, ..., *n*_m_, and the corresponding fatigue life is represented as *N*_1_, *N*_2_, ..., *N*_m_. Following the linear accumulation principle, the total damage to the rotary platform is calculated according to Formula 19:

$R_{\text{s}}=D_{\text{s}}=\sum_{i=1}^{m} \frac{n_{i}}{N_{i}}$ (19)

When *D*s=1, the distortion energy density of the gyration platform reaches its limit and fails.

**Concern # 3:** The current English expression need to improve and polish.

**Author response:**

Modifications have been made to address the inappropriate grammar statements in the paper.

**Concern # 4:** The figures are low resolution without scope of academic publish standards.

**Author response:**

Thank you very much for your comments. We have revised the images and increased their resolution.

**Concern # 5:** The fitting curves are low in physical recombination without enough factors illustration in engineering applications. Why it is high-order function with the abscissa? (Fig. 7 and Fig. 17)

During the data fitting process, we employed polynomial fitting and Gaussian fitting methods and selected the most suitable fitting formula. Taking Figure 17 ( Revised as Figure 18) as an example, it depicts the deformation of the gyration platform with varying lateral swing speeds. By utilizing Gaussian fitting, the following results were obtained:

$y=0.3902e^{-(\frac{x-1.426}{2.283})^{2}}$ (1)

The *R*^2^ of the formula (1) is 0.9798.

$y=0.01389e^{-(\frac{x-1.162}{0.2089})^{2}}+0.05779e^{-(\frac{x-1.968}{1.703})^{2}}$ (2)

The *R*^2^ of the formula (2) is 0.9995.

Similarly, polynomial fitting was applied to the data, revealing that this approach not only ensured an R^2^ value approaching unity but also provided a more straightforward and intuitive depiction of the relationship between the rotary platform's deformation and the lateral swing speed. We ultimately adopted polynomial fitting, and the formula obtained from the fitting is:

$y=0.0278x^{3}-0.0048x^{2}+0.000198x+0.026$ (3)

The *R*^2^ of the formula (3) is 0.9999.

**Concern # 6:** Figure 1 and Figure 2 should add the 3D model to support the agreed assumption before the raised question.

**Author response:**

According to the reviewer's comments, we have added a 3D model.

**Concern # 7:** The line diagrams in the referred figures should be normalized for a uniform style, including the font, size, line type, etc.

**Author response:**

According to the reviewer's comments, we have revised the figures to ensure that they have a a uniform style, including the font, size, line type, etc.

Once again, thank you very much for your constructive comments and suggestions which would help us both in English and in depth to improve the quality of the paper.

Best regards

Sincerely,

Zhen Tian

E-Mail:lntutian2008@126.com
