# Supplementary material for: Dynamic characteristics and fatigue life analysis of the gyration platform of roadheader
Source: PLoS One. 2024 Dec 13;19(12):e0314351. doi: 10.1371/journal.pone.0314351 (PMC11642995; doi:10.1371/journal.pone.0314351)
Supplement: Attachment — Submitted filename: Response-to-Reviewers.doc [file pone.0314351.s002.doc]

**Original Manuscript ID:** PONE-D-23-35060R1

**Original Article Title:** Dynamic characteristics and fatigue life analysis of gyration platform of roadheader

**To:** PLOS ONE

**Re:** Response to reviewers

Dear Editor and reviewers,

Thank you for your letter and the reviewers’ comments on our manuscript entitled "Dynamic characteristics and fatigue life analysis of gyration platform of roadheader" (ID: PONE-D-23-35060R1). Those comments are very helpful for revising and improving our paper, as well as the important guiding significance to other research. We have studied the comments carefully and made corrections which we hope meet with approval. The main corrections are in the manuscript and the responds to the reviewers’ comments are as follows .

Replies to the reviewers’ comments:

**Reviewer#2**

Special thanks to you for pointing out some important modifications needed in the manuscript. We have revised the manuscript according to your kind advises .

**Concern # 1:** Deformation trend of the gyration platform in Fig 16 is unscientific because of the missed quantitative information, neither the legend, lined maps nor comparison.

**Author response:**

Thank you very much for your comments. We have revised Fig 16 by adding legend and color scale to better illustrate the deformation trend of gyration platform.

**Concern # 2:** Some figures (e.g. Fig 17~Fig 20, Fig.22) are lacking in depth for optimized significance and designed objects

**Author response:**

Thank you very much for your comments. From Fig.17-Fig.22, we can observe the deformation and stress distribution of the gyration platform, and demonstrate the relationship between deformation and stress with the swing speed. As shown in the figures, we can see that the maximum deformation of the gyration platform mainly occurs at the ear connecting the gyration platform and the lifting hydraulic cylinder, and the maximum stress area is mainly distributed around the bolt holes of the rotary bearings. The above analysis results provide a certain reference for the structural optimization of the gyration platform and the selection of rotation speed. In order to further improve the reliability of the gyration platform, we have added structural improvement measures. Through Workbench, we have optimized parameters P10 (thickness of the two outer rib plates ), P2 (thickness of the two inner rib plates), P5 (thickness of the upper ear connecting rib plates), and P11 (radius of the bolt hole connected to the rotary bearing), as shown in Figure 24.

**The revisions are as follows:**

The locations with high stress on the gyration platform are distributed at the bolt holes connected to the rotary bearings at the front of the gyration platform, the middle part of the connecting rib plate of the ear connected to the rotary hydraulic cylinder, the ear connected to the lifting hydraulic cylinder, and the ear connected to the cutting motor box. As shown in Figure 22, these locations are also areas with lower fatigue life. In order to reduce the maximum stress of the gyration platform during the cutting process and improve its fatigue life, the parameters P10 (thickness of the two outer rib plates FD1), P2 (thickness of the two inner rib plates), P5 (thickness of the upper ear connecting rib plates), and P11 (half diameter of the bolt hole connected to the rotary bearing) were optimized, as shown in Figure 24.

**Fig 24.** Optimization parameters of gyration platform

According to the design dimensions of the cutting machine case and the relative position between the body frame and the hydraulic cylinder, the variation range of parameters P10, P2, and P5 is 25-30mm, and the variation range of parameter P11 is 8.1-9.9mm. The optimization design objective parameters are to minimize the increase in mass of the gyration platform and reduce the maximum stress of the gyration platform. Figure 25 shows the optimized design nodes of different parameters obtained from simulation analysis.

**Fig 24.** Optimized design nodes of different parameters

20 sampling points for optimized design were determined based on the numerical range of design parameters, and the MOGA method was selected for optimization based on model characteristics. The simulation provides three optimization schemes, as shown in Table 6, and by comparing the relevant parameters in different schemes, scheme 1 was selected as the optimization scheme. The mass of the optimized model is 749.18 kg, representing a mere 9.03 kg increase compared to its pre-optimized state. Furthermore, the maximum stress in the optimized model measures 293.23 MPa, indicating a substantial reduction of 71.34 MPa from the pre-optimization stress level of 364.57 MPa. These analyses underscore a noteworthy enhancement in the stress condition of the optimized gyration platform.

**Table 6.** Results of different optimization schemes

| **NO.** | **P2 (mm)** | **P5 (mm)** | **P10 (mm)** | **P11 (mm)** | **Mass (kg)** | **Maximum stress (MPa)** |
| --- | --- | --- | --- | --- | --- | --- |
| 1 | 27.332 | 26.666 | 26.532 | 9.0594 | 749.18 | 293.23 |
| 2 | 26.618 | 28.297 | 26.939 | 8.5446 | 755.62 | 295.51 |
| 3 | 27.163 | 27.291 | 27.124 | 8.7714 | 753.86 | 298.73 |

**Concern # 3:** Results and Discussion section is raw and basic, especially there is no discussion and comparison analysis. Some constructive discussion is necessary for one research paper, rather than a one-plank narrative story.

**Author response:**

Thank you very much for your comments, We have revised the conclusion.

**The revisions are as follows:**

Based on the analysis of individual cutting pick forces, a transformation of all cutting forces to the centroid of the cutting head was executed via coordinate transformation. This methodology effectively centralized the forces, facilitating a comprehensive analysis of the instantaneous loads acting on the cutting head under diverse lateral swing conditions. The resultant loads served as external excitations for the dynamic simulation of the cutting unit model.

Noteworthy insights were gleaned through dynamic simulation analysis of the rigid-flexible coupled multibody model of the roadheader's cutting part. It was discerned that the vibration acceleration of the cutting head in the Z direction is greater than the other two directions. The connecting ear of nodes 62796 and 62797 on the gyration platform vibrates the most violently, and the vibration acceleration in the Y direction is greater than the other two directions. At the same time, this position is also the location where the gyration platform undergoes significant deformation. The gyration platform experiences a maximum stress of 258.71MPa during right-to-left lateral swinging cutting, while during left-to-right lateral swinging cutting, the maximum stress escalates to 364.57MPa. It was discerned that, during left-to-right swing cutting, the gyration platform experienced markedly elevated maximum stress in comparison to right-to-left swing cutting. This heightened stress was predominantly localized in the vicinity of the bolt holes interconnecting the gyration platform and the rotary bearings. The fatigue life analysis shows that the node with the shortest fatigue life is mainly located in the front area of the rotating platform, near the edge of the bolt hole connecting the rotating platform and the rotating bearing. Among these nodes, node 33 exhibits the lowest fatigue life and can withstand a maximum cyclic load of 5.366×10^6^ cycles. With an escalation in swing speed, there was a conspicuous intensification in both the maximum stress and deformation of the gyration platform, consequently leading to a marked reduction in its fatigue life. It is noteworthy that, when confronted with the challenge of cutting hard rock,it is necessary to reduce the rotational speed to ensure the reliability of key components. In order to improve the reliability of the gyration platform, the relevant structural parameters of the gyration platform, such the thickness of the two outer rib plates, the thickness of the two inner rib plates, the thickness of the upper ear connecting rib plates, and the diameter of the bolt hole connected to the rotary bearing, were optimized and designed. The maximum stress of the optimized model is 293.23MPa, which is 71.34 MPa lower than the 364.57 MPa before optimization. The analysis results indicate that the stress state of the optimized gyration platform can be significantly improved.

The research findings not only furnish valuable guidance for enhancing the structural integrity of the gyration platform but also provide insights into the judicious selection of swing speeds, thereby contributing significantly to the overall enhancement of roadheader performance. In this study, we concentrated on scrutinizing the horizontal rotary motion of the roadheader. Nevertheless, during the practical cutting process, the roadheader exhibits both horizontal and vertical movements. The dynamic attributes of the roadheader exhibit variations in different directions of motion. Consequently, our study possesses certain limitations within its scope. To conduct a more comprehensive analysis of the roadheader's performance throughout the entire cutting process, our subsequent research will prioritize examining the dynamic characteristics of the roadheader during vertical motion.

**Concern # 4:** Three-lines table is welcome in the research paper.

**Author response:**

Thank you very much for your comments, We have revised the tables.

**Reviewer#3**

Special thanks to you for pointing out some important modifications needed in the manuscript. We have revised the manuscript according to your kind advises .

**Concern # 1:** “Due to the cutting power of this type of roadheader being 75kW, the swing speed should be ensured to be within 1.1m/min during the cutting process.” Please confirm if 1.1 m/min is correct？

**Author response:**

Thank you very much for your comments. According to formulas 1 and 2 (Ref : Li G. X. & Li X. H. Design of Coal Mining Machinery,Shenyang: Liaoning University Press; 1994.), the swing speed of the roadheader under the condition of maximum cutting power can be calculated. It is correct to calculate a maximum swing speed of approximately 1.1 m/min when the cutting power is 75kW.

The cutting power could be obtained:

$N_{P}=\frac{F_{n}\cdot V_{p}}{1000\eta_{p}}$ (1)

In the formula above: *V_p_* is the swing speed of the working mechanism; $\eta_{p}$ is the mechanical efficiency of the transmission device. *F_n_* is the average total cutting resistance of spiral drum, which can be obtained from the following formula:

$F_{n}=0.5K_{Y}K_{cc}\sum_{i=1}^{N_{c}} Z_{cpi}n_{i}$ (2)

In the formula above: *K_YK_* is the coefficient of influence of the thickness of the mined coal seam on the envelope angle formed by the working mechanism and the average cutting force; *K_cc_* is the cutting direction coefficient of coal seam weakening and working mechanism turning relative to coal bedding; *N_C_* is the number of tooth groups; *Z_cpi_* is the average cutting force of the i-th cut; *n_i_* is the number of cutting teeth working simultaneously.

**Concern # 2:** “The maximum relative error of the effective torque values obtained by the two methods under different conditions is 10.2929%.” Please explain whether it is reasonable for the error to reach 10.2929%, and suggest modifying the model to reduce the error.

**Author response:**

Thank you very much for your comments. By checking the model, it was found that when the swing speed from left to right is 0.9 m/min, the simulated torque value should be 1.1063, resulting in an error of 9.48%. In the cutting test, we used cement, water reducing agent, and lime to create a simulated working face for the roadheader to cut. The materials produced in this way may have certain non-uniform properties. In simulation calculations, the theoretical calculation formula is calculated based on homogeneous rock materials, which may cause errors between the two methods. However, through comparative analysis, it was found that the minimum error of the two methods is 3.66%, the maximum error is 9.48%, and the error is within 10%. Therefore, it can be considered that the simulation results can reflect the actual working situation of the roadheader.

**The revisions are as follows:**

To validate the accuracy of the load calculations, a simulated working face mirroring the properties of the rock cut by the specified roadheader was created using cement, water reducing agent, and lime. The cutting part structure of the roadheader was replicated for cutting tests on this simulated working face, as illustrated in Figure 10. The torque during the cutting process was obtained by installing a torque sensor on the cutting motor torque shaft, and the torque value was obtained using a data acquisition device.The results of tests and simulations comparing torque with varying swing speeds are presented in Figure 11 and Table 3. The maximum relative error between the effective torque values obtained through experimental and simulation methods under different conditions is 9.48%. This indicates the reliability of the load data derived from the theoretical calculations. Importantly, the calculated load data serves as a robust source of external excitation for multi-body dynamics simulations.

**Fig 11.** The error between test and simulation results

**Table 3.** Torque values obtained through test and simulation.

| Torque  ×10^7^ N·mm | Swing speed from left to right m/ min^-1^ | | | | | | Swing speed from right to left m/ min^-1^ | | | | | |
| --- | --- | --- | --- | --- | --- | --- | --- | --- | --- | --- | --- | --- |
|  | 0.6 | 0.7 | 0.8 | 0.9 | 1.0 | 1.1 | 0.6 | 0.7 | 0.8 | 0.9 | 1.0 | 1.1 |
| Test | 0.9215 | 0.9854 | 0.9977 | 1.0014 | 1.1212 | 1.1394 | 0.8341 | 0.8598 | 0.9045 | 0.9342 | 0.9741 | 0.9987 |
| Simulation | 0.9912 | 1.0278 | 1.0798 | 1.1063 | 1.1725 | 1.2021 | 0.8703 | 0.8958 | 0.9389 | 0.9712 | 1.0395 | 1.0553 |
| Error /% | 7.03 | 4.13 | 7.6 | 9.48 | 4.38 | 5.22 | 4.16 | 4.02 | 3.66 | 3.81 | 6.29 | 5.36 |

**Concern # 3:** Figure 11 shows periodic phenomenon in acceleration Y, please explain?

**Author response:**

Thank you very much for your comments. The vibration acceleration mentioned by the reviewer should be a periodic phenomenon observed in the vibration acceleration shown in Figures 13 and 15. When the cutting head cuts the rock, the number of the cutting picks involved in cutting is constantly changing, and it shows a periodic change. As a result, the load on the cutting head also changes periodically, as shown in Figure 6. when the load was added to the model and simulated, the acceleration in the three directions of the cutting head and the node 62796 of gyration platform will exhibit periodic phenomenon.

**Reviewer#4**

Special thanks to you for pointing out some important modifications needed in the manuscript. We have revised the manuscript according to your kind advises .

**Concern # 1:** The difficult or urgency for investigating the stress and fatigue life of a roadheader's gyration platform need to be explain firstly. Why does few of the existing studies involve the dynamics of the gyration platform?

**Author response:**

Thank you very much for your comments. We have added a necessity analysis for studying the stress and fatigue life of the gyration platform in the introduction section.The research on the dynamic characteristics of roadheader mainly focuses on the analysis of the forces acting on the cutting teeth and the impact of cutting teeth on the vibration of the roadheader. Many existing studies have also been conducted on the gyration platform, but static or modal analysis methods are often used, which cannot fully obtain the dynamic characteristics of the gyration platform during the cutting process of the roadheader. We found that studying the stress, deformation, and fatigue life of the gyration platform under different lateral directions and lateral velocities can help us find methods to improve the reliability of the gyration platform and thereby improve the performance of the roadheader.

**The revisions are as follows:**

These studies have confirmed the accuracy and reliability of finite element and dynamic simulation results, yielding valuable outcomes. For the roadheader, the gyration platform as a vital component, linking the main body frame and supporting the cutting arm. During the cutting process, the gyration platform needs to achieve the lifting and rotation of the cutting arm and bear strong alternating loads, which is a weak link. However, few of the existing studies have involved the dynamics of the gyration platform under various lateral swing conditions and the influence of the lateral swing speed on the fatigue performance.

**Concern # 2:** The current studies cannot well illustrate the dynamic characteristics and fatigue life of gyration platform. Only the torque applied on the gyration platform and the acceleration and stress at some nodes were discussed. These data cannot reflect the dynamic characteristics such as vibration and modality. The fatigue life was figured out from a S-N curve of common Q235A material in the software by importing the stress distribution. Whether is this computation reliable to determine the actual fatigue life of the whole gyration platform?

**Author response:**

Thank you very much for your comments. In order to better illustrate the dynamic characteristics of the gyration platform, we conducted modal analysis on the cutting part of the roadheader, obtained the first eight modal frequencies andvibration modal shape of the gyration platform, and analyzed the vibration deformation of the gyration platform under different modes.

In the process of analyzing the fatigue life of the gyration platform, we found through analyzing the relevant methods in the references literature that by introducing stress distribution, the fatigue life under different stress levels can be calculated from the S-N curve of the material in the software. References 22-23 indicate that the feasibility of this method has been validated. Through analysis, we obtained the fatigue life distribution of the gyration platform as shown in Figure 22. The fatigue life distribution can intuitively display the areas with lower fatigue life of the gyration platform, which provides a basis for our subsequent optimization analysis. We have added content on optimization analysis related to the gyration platform. By optimizing the relevant parameters of the gyration platform structure, we have reduced the stress on the gyration platform and improved its fatigue life.

**The revisions are as follows:**

By utilizing ADAMS/Vibration for modal analysis of the roadheader model, both the roadheader mode and its corresponding vibration shapes can be determined. This analysis allows for the calculation of modal natural frequencies and characteristic values for each mode, as presented in Table 5. The frequencies of the 1st to 2nd order modes are relatively small, and the corresponding vibration shapes are the upper and lower torsional deformation of the ears connecting the gyration platform and the height adjusting cylinder around the center of the gyration platform. The vibration shapes of the 3rd to 4th order modes and the 6th to 8th order modes are basically consistent with the vibration trend of the 1st to 2nd order modes. However, there are large deformations at the connection between the gyration platform and the rotary cylinder, the connecting rib plate in the middle of the left and right ears and the left and right sides of the rotary plate. In the 5th mode shape, the maximum deformation position of the gyration platform is concentrated in the ear part of the side connected with the height adjusting cylinder, and there is a certain bending at the tail of the rotary plate.

**Table 5.** Modal frequency and mode shape of the gyration platform

| **Modal order** | **Frequency**  **(Hz)** | **Modal shape** | **Modal order** | **Frequency**  **(Hz)** | **Modal shape** |
| --- | --- | --- | --- | --- | --- |
| 1 | 1.3207 |  | 5 | 113.366 |  |
| 2 | 2.6256 |  | 6 | 114.549 |  |
| 3 | 87.766 |  | 7 | 116.099 |  |
| 4 | 88.273 |  | 8 | 292.813 |  |

The locations with high stress on the gyration platform are distributed at the bolt holes connected to the rotary bearings at the front of the gyration platform, the middle part of the connecting rib plate of the ear connected to the rotary hydraulic cylinder, the ear connected to the lifting hydraulic cylinder, and the ear connected to the cutting motor box. As shown in Figure 22, these locations are also areas with lower fatigue life. In order to reduce the maximum stress of the gyration platform during the cutting process and improve its fatigue life, the parameters P10 (thickness of the two outer rib plates ), P2 (thickness of the two inner rib plates), P5 (thickness of the upper ear connecting rib plates), and P11 (diameter of the bolt hole connected to the rotary bearing) were optimized, as shown in Figure 24.

**Fig 24.** Optimization parameters of gyration platform

According to the design dimensions of the cutting machine case and the relative position between the body frame and the hydraulic cylinder, the variation range of parameters P10, P2, and P5 is 25-30mm, and the variation range of parameter P11 is 8.1-9.9mm. The optimization design objective parameters are to minimize the increase in mass of the gyration platform and reduce the maximum stress of the gyration platform.Figure 25 shows the optimized design nodes of different parameters obtained from simulation analysis.

**Fig 25.** Optimized design nodes of different parameters

20 sampling points for optimized design were determined based on the numerical range of design parameters, and the MOGA method was selected for optimization based on model characteristics. The simulation provides three optimization schemes, as shown in Table 7, and by comparing the relevant parameters in different schemes, scheme 1 was selected as the optimization scheme. The mass of the optimized model is 749.18 kg, representing a mere 9.03 kg increase compared to its pre-optimized state. Furthermore, the maximum stress in the optimized model is 293.23 MPa, indicating a substantial reduction of 71.34 MPa from the pre-optimization stress level of 364.57 MPa. These analyses underscore a noteworthy enhancement in the stress condition of the optimized gyration platform.

**Table 7.** Results of different optimization schemes

| **NO.** | **P2 (mm)** | **P5 (mm)** | **P10 (mm)** | **P11 (mm)** | **Mass (kg)** | **Maximum stress (MPa)** |
| --- | --- | --- | --- | --- | --- | --- |
| 1 | 27.332 | 26.666 | 26.532 | 9.0594 | 749.18 | 293.23 |
| 2 | 26.618 | 28.297 | 26.939 | 8.5446 | 755.62 | 295.51 |
| 3 | 27.163 | 27.291 | 27.124 | 8.7714 | 753.86 | 298.73 |

**Concern # 3:** In the sentence 'the cutting picks experience forces as illustrated in Figure 1', Figure 1 should be Figure 2.

**Author response:**

Thank you very much for your comments. According to the reviewer's comments, we have revised the content.

**Concern # 4:** Fig.16 should be presented at a more appropriate view angle to better illustrate the deformation. In addition, what does the color in this figure indicate? A color scale should be added next to the model.

**Author response:**

Thank you very much for your comments. We have revised Fig 16 by adding legend and color scale to better illustrate the deformation trend of gyration platform.

**Concern # 5:** What does the deformation in Fig.18 stand for? Where is this deformation?

**Author response:**

Thank you very much for your comments. The deformation in Figure 18 refers to the maximum deformation of the gyration platform when it swings from left to right under different swing speed conditions. Due to the different swing speeds, the position of the node where the maximum deformation occurs will also change. Through analysis of the deformation of the gyration platform at different swing speeds, it was found that the maximum deformation mainly concentrates on the ear connecting the gyration platform and the height adjustment hydraulic cylinder.

**Concern # 6:** What does the fatigue life in Fig.23 stand for? I just wonder what does the minimum fatigue life mean? If the nodes within the gyration platform had diffferent fatigue life, what should be the fatigue life of this component, i.e. gyration platform?

**Author response:**

Thank you very much for your comments. The fatigue life in Figure 23 refers to the minimum fatigue life of a node on the gyration platform obtained through analysis. If the fatigue life of a node is the minimum, it indicates that the position is most prone to fatigue failure. In reviewing relevant fatigue life analysis references, it is proposed that the node with the lowest fatigue life and the occurrence of fatigue damage can be considered as the fatigue life of the corresponding parts. Although the nodes in the gyration platform have different fatigue lives, the fatigue life distribution shown in Figure 22 shows that the positions of nodes with low fatigue life are concentrated, mainly located at the connection hole between the gyration platform and the rotary bearing. Therefore, it is feasible to use the lowest fatigue life of a node as the fatigue life of the gyration platform for subsequent structural optimization and selection of motion parameters.

Once again, thank you very much for your constructive comments and suggestions which would help us both in English and in depth to improve the quality of the paper.

Best regards

Sincerely,

Zhen Tian

E-Mail:lntutian2008@126.com
