# Supplementary material for: Dynamic characteristics and fatigue life analysis of the gyration platform of roadheader
Source: PLoS One. 2024 Dec 13;19(12):e0314351. doi: 10.1371/journal.pone.0314351 (PMC11642995; doi:10.1371/journal.pone.0314351)
Supplement: Attachment — Submitted filename: Response-to-Reviewers.doc [file pone.0314351.s003.doc]

**Original Manuscript ID:** PONE-D-23-35060R2

**Original Article Title:** Dynamic characteristics and fatigue life analysis of gyration platform of roadheader

**To:** PLOS ONE

**Re:** Response to reviewers

Dear Editor and reviewers,

Thank you for your letter and the reviewers’ comments on our manuscript entitled "Dynamic characteristics and fatigue life analysis of gyration platform of roadheader" (ID: PONE-D-23-35060R2). Those comments are very helpful for revising and improving our paper, as well as the important guiding significance to other research. We have studied the comments carefully and made corrections which we hope meet with approval. The main corrections are in the manuscript and the responds to the reviewers’ comments are as follows .

Replies to the reviewers’ comments:

**Reviewer#4**

Special thanks to you for pointing out some important modifications needed in the manuscript. We have revised the manuscript according to your kind advises .

**Concern # 1:** The manuscript has been revised carefully. But I still wonder the academical significance for investigating fatigue life of a roadheader's cutting head and gyration platform. Neither innovative methods nor valuable conclusions were proposed. In addition, I don't think the fatigue life has been well examined in this study, because the 'fatigue life' was just given from a certain stress according to S-N curves of materials. It means the original data were still stress, i.e. the discusses were still stress analysis. Only difference is that they were explained from another view by assigning them to a certain fatigue life.

Nevertheless, this manuscript presents with relative high scholarly format and thus I'm not opposed to accepting it after minor modification. The conclusion need to be revised. It's too verbose to well refelct the objective and some main contribution of this study.

**Author response:**

Thank you very much for your comments.

The roadheader will be subjected to nonlinear impact loads during the process of cutting rocks, which will have adverse effects on the reliability of the key mechanical structure of the roadheader. Nonlinear impact load is the result of the cutting teeth on the cutting head breaking the rock, so studying the impact load on the cutting head is a prerequisite for studying the reliability and fatigue life of the mechanical structure of the roadheader. For a roadheader, the gyration platform is an important component that connects the main frame and supports the cutting arm. During the cutting process, the gyration platform needs to achieve the lifting and rotation of the cutting arm and withstand strong alternating loads, which is a weak link. If the gyration platform is damaged, it will affect the normal progress of excavation work, as shown in Figure 1. Therefore, the reliability and fatigue life of the gyration platform are crucial for the normal operation of the roadheader. On the basis of studying the impact load of the cutting head under different working conditions, this paper comprehensively analyzes the dynamic characteristics of the gyration platform in different swing directions and swing speeds, such as vibration acceleration, stress, strain, and modal analysis. Combined with Miner’s fatigue damage theory, the fatigue life of the gyration platform at different swing speeds and swing directions was evaluated. Based on the analysis results, the structure of the gyration platform was optimized. The methods and results adopted in the study are of great significance for the structural design of the gyration platform and the reasonable selection of the swing speed of the roadheader.

Fig 1. Damaged gyration platform

The gyration platform operates in a highly complex vibration environment, where various parts of its structure are subjected to stress and strain that change over time. Studying the fatigue life of the gyration platform is to investigate the number of stress cycles experienced by the gyration platform before fatigue failure. The commonly used fatigue life estimation method in engineering currently involves first determining the external load and obtaining the S-N curve of the material or component through testing to describe the relationship between the number of fatigue fracture cycles and cyclic stress that the material is subjected to. At the same time, the Miner's fatigue damage theory was used to estimate the fatigue life of the gyration platform. The Miner's fatigue damage theory has the characteristics of fast calculation speed, easy to understand solution process, and in most cases, its estimated life value is quite close to the experimental results. Therefore, the Miner's fatigue damage theory was used in this study. In Miner's fatigue damage theory, the stress of the gyration platform is the basis for analyzing fatigue life. Only by determining the stress of the gyration platform and establishing an accurate load spectrum can the accurate analysis of the fatigue life of the gyration platform be achieved.

According to the reviewer's comments, we have revised the conclusion.

**The revisions are as follows:**

Based on the analysis of individual cutting pick forces, a transformation of all cutting forces to the centroid of the cutting head was executed via coordinate transformation. This methodology effectively centralized the forces, facilitating a comprehensive analysis of the instantaneous loads acting on the cutting head under diverse lateral swing conditions. The resultant loads served as external excitations for the dynamic simulation of the cutting unit model.

Noteworthy insights were gleaned through dynamic simulation analysis of the rigid-flexible coupled multibody model of the roadheader's cutting part. During left-to-right swing cutting, the gyration platform experienced markedly elevated maximum stress in comparison to right-to-left swing cutting. This heightened stress was predominantly localized in the vicinity of the bolt holes interconnecting the gyration platform and the rotary bearings. The fatigue life analysis shows that the node with the shortest fatigue life is mainly located in the front area of the rotating platform, near the edge of the bolt hole connecting the rotating platform and the rotating bearing. With an escalation in swing speed, there was a conspicuous intensification in both the maximum stress and deformation of the gyration platform, consequently leading to a marked reduction in its fatigue life. It is noteworthy that, when confronted with the challenge of cutting hard rock,it is necessary to reduce the swing speed to ensure the reliability of key components.

In order to improve the reliability of the gyration platform, the relevant structural parameters of the gyration platform, such the thickness of the two outer rib plates, the thickness of the two inner rib plates, the thickness of the upper ear connecting rib plates, and the diameter of the bolt hole connected to the rotary bearing, were optimized and designed. The analysis results indicate that the stress state of the optimized gyration platform can be significantly improved. The research findings not only furnish valuable guidance for enhancing the structural integrity of the gyration platform but also provide insights into the judicious selection of swing speeds, thereby contributing significantly to the overall enhancement of roadheader performance.

Once again, thank you very much for your constructive comments and suggestions which would help us improve the quality of the paper.

Best regards

Sincerely,

Zhen Tian

E-Mail:lntutian2008@126.com
