# Supplementary material for: Dynamic characteristics and fatigue life analysis of the gyration platform of roadheader
Source: PLoS One. 2024 Dec 13;19(12):e0314351. doi: 10.1371/journal.pone.0314351 (PMC11642995; doi:10.1371/journal.pone.0314351)
Supplement: Attachment — Submitted filename: Response-to-Reviewers.doc [file pone.0314351.s004.doc]

**Original Manuscript ID:** PONE-D-23-35060R3

**Original Article Title:** Dynamic characteristics and fatigue life analysis of gyration platform of roadheader

**To:** PLOS ONE

**Re:** Response to reviewers

Dear Editor and reviewers,

Thank you for your letter and the reviewers’ comments on our manuscript entitled "Dynamic characteristics and fatigue life analysis of gyration platform of roadheader" (ID: PONE-D-23-35060R3). Those comments are very helpful for revising and improving our paper, as well as the important guiding significance to other research. We have studied the comments carefully and made corrections which we hope meet with approval. The main corrections are in the manuscript and the responds to the reviewers’ comments are as follows .

Replies to the reviewers’ comments:

**Reviewer#6**

Special thanks to you for pointing out some important modifications needed in the manuscript. We have revised the manuscript according to your kind advises .

**Concern # 1:**

1. Formula (1) – (12) should be provided with units. How is the term “width of the pick blade” (formula (1)) understood if the cutting head is equipped with conical picks? Where is the pick width measured and how much is it – the tip diameter (Alloy head diameter) is given in Tab. 2)?

**Author response:**

Thank you very much for your comments. We have added the units in the parameter descriptions below each formula. Based on the comments from the reviewers, we have revised the meaning of the relevant parameters in Formula 1. When the cutting head using flat picks, the parameter $k_{b}$ is required, and the accurate meaning of $k_{b}$ is the influence coefficient of the blade width of the flat pick. When cutting head using conical picks, the geometric shape influence coefficient $k_{g}=k_{\psi}\cdot k_{\psi'}\cdot k_{d}$. At this time, the influence coefficient of the cutting width of the cutting pick is not required to calculate the force of the cutting pick, but the influence coefficient of alloy head diameter is required to calculate the geometric shape influence coefficient $k_{g}$. The influence coefficient of alloy head diameter $k_{d}$can be interpolated based on the data in Table 1.

Table 1. The Influence coefficient of alloy head diameter

| Alloy head diameter | 9 | 12 | 14 | 16 |
| --- | --- | --- | --- | --- |
| $k_{d}$ | 1 | 1.11 | 1.26 | 1.4 |

According to the coal breaking theory, the diameter of the alloy head $d$ and the diameter of pick shank $D$ can be measured according to the following Fig. a, where the calculated working width of the cutting pick is half of the diameter of pick shank.

**Fig. a** The diameter of the alloy head and the diameter of pick shank

**The revisions are as follows:**

（1）The cutting resistance $Z$ could be obtained:

$Z=p_{k}\left[k_{T}\cdot k_{\text{g}}\cdot k_{\text{y}}\left(0.25+0.018t\cdot h\right)+0.1s_{\text{z}}\right]$ （1）

The traction resistance $Y$could be obtained:

$Y=k_{n1}\cdot Z$ （2）

The lateral force $X$ could be obtained:

$X=k_{n2}\cdot Z$ （3）

In the formula above:$Z$is the cutting resistance of a cutting pick, in N;$Y$is the traction resistance of a cutting pick, in N;$X$is the lateral force of a cutting pick, in N;$p_{k}$ is rock contact strength, in MPa; $k_{\text{T}}$ is the cutting pick type coefficient; $k_{g}$is coefficient of influence on pick geometry, for conical pick:$k_{g}=k_{\psi}\cdot k_{\psi'}\cdot k_{d}$,$k_{\psi}$ is the shape coefficient of the alloy head, $k_{\psi'}$ is the shape coefficient of the cutting pick head, $k_{d}$is the influence coefficient of alloy head diameter, for flat pick:$k_{g}=k_{q}\cdot k_{b}$,$k_{q}$is the influence coefficient of cutting pick front edge shape, $k_{b}=0.92+0.01b$, *b* is the width of the pick shank, in mm; $k'_{\text{y}}$ is the influence coefficient of the cutting angle of the cutting pick; $t$ is the mean value of intercept distance, in mm;$h$is the average value of chip thickness, in mm; $s_{\text{z}}$ is the projected area in the traction direction after blunting the rear edge surface, in mm^2^; $k_{n1}$ is the coefficient of influence of cutting resistance on traction resistance;$k_{n2}$is the coefficient of influence of cutting resistance on lateral force.

(2) The resultant force in the X direction on each cutting line could be obtained:

(4)

The traction resistance in the Y direction on each cutting line could be obtained:

$R_{yi}=\sum_{j=1}^{n} (-Y_{ij}\sin\theta-Z_{ij}\cos\theta)$ (5)

The lateral force in the Z direction on each cutting line could be obtained:

$R_{zi}=\sum_{j=1}^{n} X_{ij}$ (6)

In the formula above,$R_{xi}$is the resultant force of the cutting picks on each cutting line, in N; $R_{yi}$is the traction resistance of the cutting picks on each cutting line, in N; $R_{zi}$ is the lateral force of the cutting picks on each cutting line, in N; $i$ is the *i*-th section;$j$ is the *j-*th pick on each section line; $n$ is the total number of picks on each section line; $N$ is the number of cut lines; $X_{ij}$is the lateral force of the *i*-th pick on the *j*-th section line, in N; $Y_{ij}$ is the traction resistance of the *i*-th pick on the *j*-th section line, in N; $Z_{ij}$ is the resultant force of the *i*-th pick on the *j*-th section line, in N; $\theta$ is the position angle of the pick, in °. Convert the force on all cutting picks on each cutting line to the centroid of the cutting head, and the resulting three-dimensional force and torque can be computed using formulas (7) to (12).

The resultant force of the cutting head in the X direction could be obtained:

$R_{x}=\sum_{i=1}^{N} R_{xi}$ (7)

The traction resistance of the cutting head in the Y direction could be obtained:

$R_{y}=\sum_{i=1}^{N} R_{yi}$ (8)

The lateral force of the cutting head in the Z direction could be obtained:

$R_{z}=\sum_{i=1}^{N} R_{zi}$ (9)

The moment of the cutting head in the X direction could be obtained:

$M_{x}=\sum_{i=1}^{N} R_{yi}\cdot d_{i}+\sum_{i=1}^{N} 500R_{zi}\sin\theta\cdot D+\frac{3}{4}R_{s}\cos\alpha\cdot D\cdot500$ (10)

The moment of the cutting head in the Y direction could be obtained:

$M_{y}=-\sum_{i=1}^{N} R_{xi}\cdot d_{i}+\sum_{i=1}^{N} 500R_{zi}\cos\theta\cdot D+\frac{3}{4}R_{s}\cos\alpha\cdot D\cdot500$ (11)

The moment of the cutting head in the Z direction could be obtained:

$M_{z}=\sum_{i=1}^{N} \sum_{j}^{n} Z_{ij}D\cdot500$ (12)

In the formula above: $R_{x}$is the resultant force of the cutting head, in N;$R_{y}$is the traction resistance of the cutting head, in N;$R_{z}$is the lateral force of the cutting head, in N;$M_{x}$is the moment of the cutting head in the X direction, in N·mm;$M_{y}$is the moment of the cutting head in the Y direction, in N·mm;$M_{z}$is the moment of the cutting head in the Z direction, in N·mm;$\theta$ is the position angle of the pick, in °; $\alpha$ is the friction angle between blade and rock, in °; $d_{i}$ is the distance between the *i*-th section and the centroid, in mm; $D$ is the cutting head diameter at the location of the cutting pick, in mm.

**Concern # 2:**

2. What are the values given in formulas (17) and (18) (187.9 mm and 2.2 mm/s), on what basis were they calculated, since no data is given?

**Author response:**

Thank you very much for your comments. By checking the model, we have added relevant data, which can be input into formula 17 to calculate the elongation of the hydraulic cylinder. The elongation of the hydraulic cylinder was calculated to be 187.9mm. According to the swing model shown in Figure 5, it can be calculated that when the maximum swing speed is 1.1m/min, the extension and contraction speed of the hydraulic cylinder is 2.2mm/s.

**We have revised the corresponding content of the manuscript. The revisions are as follows:**

(1) According to the structure and actual working conditions of this type roadheader, $AA'$=332.9mm, $AB$=646.7mm, $OC$=3000mm,$L$=1500mm, $\alpha$=30°, $\beta$=58.3°, $\gamma$=54.6°. The elongation of the hydraulic cylinder was calculated to be 187.9mm.

Based on the operational efficiency stipulated for the roadheader, the cutting time required for the yaw cutting can be computed. Subsequently, the extension speed of the hydraulic cylinder is determined by:

$v_{1}=\frac{Exl}{t}$ (18)

(2) Operating at full load, the maximum transverse swing speed of the cutting head is approximately 1.1 m/min. At this time, the expansion and contraction speed of the hydraulic cylinder is 2.2mm/s.

**Concern # 3:**

3. Table 2 – the pick shank diameter and alloy head diameter seem to be unnecessary from the point of view of the presented analyses.

**Author response:**

Thank you very much for your comments. Based on the response to comment # 1 and the explanation of Formulas 1-3, when the cutting head equipped with conical picks, the calculation of the force on the cutting pick requires the diameter of the alloy head, and the diameter of the pick shank is not a necessary parameter. According the reviewer's comments, we have revised Table 2.

**Concern # 4:**

4. The description of Fig. 7-9 states that with the increase in boom swing speed, the torque and power consumption for cutting increase. Meanwhile, Fig. 9 shows that the cutting torque decreases. Where in Fig. 7 and 8 is the increase in load and power visible – the courses have a periodic character with a rotation period of the cutting head (1.13 s)?
   **Author response:**

Thank you very much for your comments. Fig. 7 and Fig. 8 shows the torque and power variation curves of the cutting head at a swinging speed of 1.1m/s. Due to the fact that the roadheader starts cutting rocks at 0.3s, the torque and power consumption of the cutting head increase sharply from 0.3s. Due to the rotation of the cutting head, the number of cutting teeth involved in cutting rocks on the cutting head is constantly changing. Therefore, the fluctuation of the curve in Fig.7 and Fig. 8 has a certain periodicity, with a fluctuation period of about 1.13s. Figure 9 shows the changes in torque and power under different swing speed conditions.

**Concern # 5:**

5. The last line of the paragraph under Fig. 6 refers to optimal mining conditions. This is a rather lofty formulation, and it is not clear what it means.

**Author response:**

Thank you very much for your comments. The cutting motor power of this roadheader is 75 kW, and the rotation speed of the cutting head is 53 r/min. Based on the measurement results of rock properties, it is calculated and analyzed that the maximum transverse swing speed of the cutting head is approximately 1.1 m/min. During the cutting process, the swing speed must be kept within 1.1 m/min to ensure that the machine is not overloaded. According to the reviewer's comments, we have revised this sentence to make it more accurate.

**Concern # 6:**

6. In the description of the directions of acceleration components in the paragraph under Fig. 13, the Z axis is the vertical direction, and the X axis is the horizontal direction. This is different from Figs. 2 and 3. This is confusing.
**Author response:**

Thank you very much for your comments. In the force analysis of cutting pick, it is customary to name cutting resistance *F_z_*, traction resistance *F_y_*, and lateral force *F_x_*. Therefore, according to the different subscripts of the forces, the direction description of the coordinate system in force analysis is often consistent with the subscripts. In this manuscript, When adding the loads as excitation in dynamic simulation, we have already adjusted the direction of the loads based on the model's coordinate system direction in the software. The Z direction in Fig.13 is consistent with the X direction in Fig. 2, as shown in Fig. b. Therefore, although the coordinate systems in Fig. 13, Fig. 2 and Fig. 3 are different, the data in the corresponding directions are correct. According to the reviewer's comments, in order to ensure consistency between the two coordinate systems, we have adjusted the coordinate system of the simulation model, as shown in Fig. c, and the adjusted coordinate system is consistent with the coordinate systems in Fig. 2 and Fig. 3, the data in Fig. 13, Fig. 15, and Table 4 have been revised.

**Fig.** b The coordinate system of the original model

**Fig.** c The coordinate system of the revised model

**Concern # 7:**
7. How were the curves shown in Figs. 13, 15, 17, and 20 obtained – is this the result of computer simulation with the excitation shown in Fig. 6?
**Author response:**

Thank you very much for your comments. Based on the force calculation of the cutting pick and the force transformation of cutting head, we can calculate the loads of the cutting head with different motion conditions through a simulation program, as shown in Fig. 6. In the dynamic software ADAMS, these loads were added as external excitations to the centroid of the cutting head in the dynamic simulation of the roadheader. Through dynamic simulation, the vibration characteristics of the cutting head and the flexible gyration platform, the stress-strain information of the flexible gyration platform can be obtained. The vibration acceleration in Fig.13 and Fig.15, the deformation in Fig.17, and the stress variation in Fig. 20 were obtained through dynamic simulation.

**Concern # 8:**

8. Table 4 – there is “Rsm” – it should be “RMS”.
**Author response:**

Thank you very much for your comments. We have revised “Rsm ”to “RMS”.

**Concern # 9:**
9. Fig. 21 – for clarity, the lines could be made in different colors.

**Author response:**

Thank you very much for your comments, We have revised the Fig. 21 and changed the colors of the lines in Figure.

Once again, thank you very much for your constructive comments and suggestions which would help us improve the quality of the paper. We hope that these revisions fulfill the requirements to make the manuscript acceptable for publication in PLOS ONE.

Best regards

Sincerely,

Zhen Tian

E-Mail:lntutian2008@126.com
